# Recent advances in inflammatory bowel disease

**Published:** 2015

**Authors:** David Al Dulaimi

**Affiliations:** *Department of Gastroenterology, Alexandra Hospital, Redditch, UK*

*Nakarai A et al* *.* ***Prognosis of ulcerative colitis differs between patients with complete and partial mucosal healing, which can be predicted from the platelet count.*** *World J Gastroenterol 2014; 20(48): 18367-18374*

The degree of mucosal healing following a flare of ulcerative colitis (UC) may provide useful prognostic information. This retrospective cohort study of 169 UC patients with sustained clinical remission (≥6 months) investigated the prognosis of complete (Mayo endoscopic score (MES) 0, n=80) and partial (MES 1, n=89) colonic mucosal healing. Patients with MES 1 had a significantly higher risk of clinical relapse, hospitalisation and need for rescue corticosteroids compared to patients with MES 0 (HR 8.17 95%CI 4.19-17.96 p<0.0001, HR 10.48 95%CI 1.90-195.22 p=0.0044, HR 27.31 95%CI 5.69-490.06 p<0.0001 respectively). Complete mucosal healing was associated with platelets <26 x 10^4^/μL and white cell count <5900/μL (OR 4.1 95%CI 2.15-7.99, OR 2.37 95%CI 1.19-4.81 respectively). Compared to patients with sustained MES 0 mucosal relapse from MES 0 to 1 was associated with an increase in platelets (+3.8 x 10^4^/μL vs -0.6 x 10^4^/μL, p<0.0001). The authors suggest that platelet monitoring may have a prognostic role in UC by helping to predict mucosal relapse.

*Miyake N et al.* ***Azathioprine is essential following cyclosporine for patients with steroid-refractory ulcerative colitis.*** *World J Gastroenterol 2015;21(1):254-261*

There is a need to clarify the optimal maintenance therapy following an acute exacerbation of UC in steroid-unresponsive patients from Asian populations. This retrospective cohort study evaluated the long-term prognosis of 29 Japanese patients with severe, steroid-refractory UC who were managed with cyclosporine from 1997 to 2008. Following cyclosporine induction patients were commenced on maintenance therapy with a combination of oral cyclosporine, oral prednisolone, tacrolimus, azathioprine, 5-aminosalicylate and leukocytapheresis. 19 patients (65.5%) exhibited a positive clinical response to cyclosporine which was sustained for at least five years in 60%. A significant association was observed between maintenance therapy including azathioprine and the long term effectiveness of cyclosporine therapy (p=0.0014). No patients had to stop maintenance therapy due to side effects although a small proportion required azathioprine dose reduction due to leucopenia and liver dysfunction. 90% of cyclosporine non-responders required surgery within six months. Overall the inclusion of azathioprine in maintenance therapy may enhance the long term efficacy of cyclosporine induction therapy in Japanese patients with severe, steroid-unresponsive UC. This should be evaluated by further larger studies.

*Sandborn WJ et al.* ***Budesonide foam induces remission in patients with mild to moderate ulcerative proctitis and ulcerative proctosigmoiditis.*** *Gastroenterology 2015;148 (4):740-750.*

The effectiveness of topical corticosteroids in the management of active distal UC including ulcerative proctitis (UP) and ulcerative proctosigmoiditis (UPS) is unclear. This study consisting of two randomised, double-blind, placebo controlled trials evaluated the efficacy of budesonide foam in inducing remission in 546 patients with mild-moderate UP or UPS. Budesonide foam administered at 2mg/25mL twice daily for two weeks and then daily for four weeks was associated with a significantly greater rate of remission, resolution of rectal bleeding and endoscopic improvement at week six compared with placebo (p<0.05). Furthermore the therapy was well tolerated and there were no cases of clinically symptomatic adrenal insufficiency. Overall rectal budesonide foam is an effective, convenient therapy for mild-moderate UP and UPS. 

*Kawakami K et al.* ***Effects of oral tacrolimus as a rapid induction therapy in ulcerative colitis.*** *World J Gastroenterol 2015;21(6): 1880-1886*

To achieve rapid remission of UC there is great clinical need for effective oral therapies which avoid the significant adverse effects of corticosteroids. This prospective, multicentre, observational study investigated the efficacy and safety of rapid induction therapy with oral tacrolimus in 49 steroid-refractory (n=38) or steroid-dependent (n=13) UC patients. Tacrolimus dosing was adjusted to maintain trough levels of 10-15 ng/mL for the first two weeks. Tacrolimus therapy was well tolerated with a high clinical response rate (73.1% at two weeks) and clinical remission rate (75.6% at four weeks). Three tacrolimus refractory patients required colectomy. Although this study suggests rapid induction with oral tacrolimus is a safe, effective treatment for refractory UC it is important to evaluate long term outcomes with future studies*.*

*Hosseini SV et al.* ***Fecal calprotectin is an accurate tool and correlated to Seo index in prediction of relapse in Iranian patients with ulcerative colitis.*** *Iran Red Crescent Med J 2015;17(2):e22796*

Fecal calprotectin (FC) is a useful non-invasive marker of intestinal inflammation but its correlation with UC severity indices such as the Seo colitis activity index is poorly understood. This prospective cohort study conducted in 154 UC patients registered in Fars province's IBD registry centre, Shiraz, Iran evaluated the correlation between FC and the Seo colitis activity index. 48.1% of UC patients relapsed during the follow-up period from 2012 to 2013. There was a significant difference in the concentration of FC between relapsing and non-relapsing patients and FC concentrations significantly correlated with the Seo activity index (p<0.001). Of note FC concentrations ≥341 μg/g were sensitive and specific for predicting relapse (sensitivity = 80%, specificity = 89%, positive predictive value = 86.76%, negative predictive value = 82.56%, positive likelihood ratio = 7.09, negative likelihood ratio = 0.23). Alongside clinical evaluation FC measurement appears to be a useful, non-invasive test to evaluate the risk of relapse in Iranian UC patients.

*Navaneethan U, Rai T, Venkatesh PGK, Kiran RP.* ***Primary sclerosing cholangitis and the risk of colon neoplasia in patients with Crohn's colitis*** *. Gastroenterology Report 2015:1-6*

It is unclear whether primary sclerosing cholangitis (PSC) increases the risk of colon cancer/dysplasia in patients with crohn's colitis (CC). This case-control cohort study of 43 adult patients with CC and PSC attending the Cleveland Clinic, Cleveland, USA between 1985 and 2015 found that concurrent PSC does not increase the risk of colon cancer/dysplasia in patients with CC. 16.3% of patients with CC and PSC compared with 14.8% of CC controls developed colon cancer or dysplasia. This relatively high rate of neoplasia/dysplasia may reflect referral bias generated by recruitment of the study population from a sub-speciality tertiary centre. Colonic neoplasia/dysplasia developed more commonly proximal to the splenic flexure in patients with CC and PSC compared to patients with CC alone (100% vs 50%, p=0.001). In both the study and control groups an increased colonic neoplasia risk was associated with male gender (p=0.008) and advancing age at CC diagnosis (p<0.001) whilst azathioprine/6-mercaptopurine use was protective (p=0.005).

*Adedokun et al.* ***Association between serum concentration of infliximab and efficacy in adult patients with ulcerative colitis*** *. Gastroenterology 2014;147:1296-1307*

Therapeutic infliximab monitoring may improve outcomes in the management of UC particularly in patients with poor response due to inadequate drug concentrations. This study analysed data from the active UC trials 1 and 2 (ACT-1 and ACT-2) post-hoc to assess the relationship between serum infliximab concentrations and clinical outcomes in 728 patients with moderately-to-severely active UC (MAYO score 6-12). Significantly greater median serum infliximab concentrations were associated with improved clinical response and mucosal healing at weeks 8, 30 and 54 and with clinical remission at weeks 30 and 54 regardless of the induction and maintenance doses (5 mg/Kg or 10 mg/Kg). For clinical response at week 8 a threshold infliximab concentration of 41 μg/mL was associated with a sensitivity, specificity and positive predictive value of 63%, 62% and 80% respectively. Furthermore to maintain clinical remission at week 30 a threshold infliximab concentration of 3.7 μg/mL was associated with a sensitivity, specificity and positive predictive value of 65%, 71% and 82% respectively.

This study highlights the possible merits of targeted infliximab dosing according to serum drug concentrations. There is a need for prospective studies to further define the optimal serum infliximab concentrations to guide the management of patients with moderately-to-severely active UC.

**Papers were prepared by:**

**Drs Luke Materacki **and** Ishfaq Ahmad, **Alexandra Hospital, UK

